# A Multichannel Strain Measurement Technique for Nanomodified Smart Cement-Based Sensors in Reinforced Concrete Structures

**DOI:** 10.3390/s21165633

**Published:** 2021-08-21

**Authors:** Andrea Meoni, Antonella D’Alessandro, Massimo Mancinelli, Filippo Ubertini

**Affiliations:** Department of Civil and Environmental Engineering, University of Perugia, Via G. Duranti, 93, 06125 Perugia, PG, Italy; andrea.meoni@unipg.it (A.M.); massimo.mancinelli@unipg.it (M.M.); filippo.ubertini@unipg.it (F.U.)

**Keywords:** smart materials, smart structures, smart cement-based sensors, carbon nanotubes, strain measurement, structural health monitoring, reinforced concrete structures, measurement techniques

## Abstract

Nanomodified smart cement-based sensors are an emerging self-sensing technology for the structural health monitoring (SHM) of reinforced concrete (RC) structures. To date, several literature works demonstrated their strain-sensing capabilities, which make them suited for damage detection and localization. Despite the most recent technological improvements, a tailored measurement technique allowing feasible field implementations of smart cement-based sensors to concrete structures is still missing. In this regard, this paper proposes a multichannel measurement technique for retrieving strains from smart cement-based sensors embedded in RC structures using a distributed biphasic input. The experiments performed for its validation include the investigation on an RC beam with seven embedded sensors subjected to different types of static loading and a long-term monitoring application on an RC plate. Results demonstrate that the proposed technique is effective for retrieving time-stable simultaneous strain measurements from smart cement-based sensors, as well as for aiding the identification of the changes in their electrical outputs due to the influence of environmental effects variable over time. Accordingly, the proposed multichannel strain measurement technique represents a promising approach for performing feasible field implementations of smart cement-based sensors to concrete structures.

## 1. Introduction

Although not yet available in the market and still in their development stages, nanomodified smart cement-based sensors [1,2] have the potential to represent a low cost and durable alternative to more traditional strain-sensing devices used for the structural health monitoring (SHM) of reinforced concrete (RC) structures, such as resistive strain-gauges and fiber optic sensors (the interested reader is referred to reference [3] for an extensive state-of-the-art on SHM technologies). They could be adopted as a potential solution for distributed sensing in concrete elements or structures, can be produced with similar procedures to those used for casting normal concrete materials, and could be easily embedded at critical structural locations. Moreover, cement-based sensors possess similar durability as the concrete structures to be monitored and have lower maintenance costs with respect to traditional sensors [4,5,6]. Produced by enhancing the piezoresistive capability of the cementitious construction materials through the addition of suitable electrically conductive fillers, smart cement-based sensors are characterized by a sensing principle based on the correlation between changes in their strain states and variations in their electrical outputs, such as their electrical resistance, current, and impedance [7,8,9,10].

Nowadays, extensive efforts have been made by the research community for the selection of the most performing fillers, among which carbon-based fillers, such as graphene nanoplatelets (GNP), carbon nanofibers (CNF)s, carbon nanotubes (CNT)s, and multiwalled carbon nanotubes (MWCNT)s, appear particularly suitable [11,12,13,14]. Several investigations proving the effectiveness of smart cement-based sensors for monitoring strain under loads of both static and dynamic nature can be found in literature [15,16,17,18], including studies demonstrating their capability to enable damage detection and localization when embedded or externally anchored in concrete structural elements [19,20,21,22,23].

Despite the fairly mature level achieved by smart cement-based sensors, the development of measuring techniques allowing their feasible field implementations to RC structures is still a challenge. In this context, Han et al. [24] developed a data acquisition system for carrying out voltage measurements through the DC four-pole method from carbon fiber cement paste piezoresistive sensors. In particular, the system was designed to simultaneously collect analog voltage signals from a sensor and its corresponding resistor placed in series within the circuit. The outputs are turned into digital signals through an A/D card, then processed to calculate the electrical resistance of the sensor. Multichannel acquisitions can be also performed simply by setting up different circuits consisting of a reference resistance placed in series with multiple sensors. Nevertheless, the system is prone to polarization issues, which cause a time-based drift in the measurements. Machan and Steffan [25] developed a low-cost multichannel laboratory device to perform electrical measurements from smart cement-based sensors that can also sense temperature and humidity. In this case, each independent channel refers to a sensor powered through a 1 kHz square wave AC technique, while the voltage drop at the known resistor, placed in series within the circuit, is recorded and used for computing the electrical resistance of the sensor. The device allows performing electrical measurements on eight channels at the same time, which is quite a limited number in the case of field applications of smart cement-based sensors to RC structures that usually require a more diffused monitoring. As a further drawback, it possesses heating issues that can cause temperature drift in the measurements from the reference resistors. Downey et al. [26] proposed an approach based on a similar, yet slower (typically 1 Hz), square wave input, termed biphasic DC, allowing simultaneous multichannel measurements in different sections of a strain-sensing cementitious element. Here, sections are powered with a square wave voltage input while measuring the voltage drop across each pair of electrodes to determine their electrical resistance. Each portion of the cementitious material is therefore treated as a resistor of unknown value placed in series with the others. Cracks that occur in a section can be detected by seeking for variations in the corresponding electrical outputs [27]. Although very useful in the case of bulk applications of strain-sensing cement-based materials, the biphasic approach proposed by Downey and co-authors is not suitable for discrete smart cement-based sensors embedded in different structural elements. Along these lines, Kang et al. [28] developed an instantaneous multichannel acquisition system for gathering impedance measurements from fiber-reinforced cementitious composites for crack localization. Stimulating the sensing material with an AC input of the desired waveform, the system acquires the current propagating through the composite while computing its voltage by means of a self-sensing circuit based on a capacitive voltage divider. A multiplexer is used to repeat measurements in different channels, each corresponding to a region of the sensing material. Similar to the previously proposed measurement technique, this system does not allow performing measurements from discrete independent smart cement-based sensors.

In this study, a new multichannel strain measurement technique is proposed to simultaneously retrieve time-stable strain measurements from smart cement-based sensors embedded in key sections of RC structures through the measuring and processing of their electrical outputs in a real-time fashion. The proposed technique is conceived to keep the best feature of the methods reviewed above, while also overcoming their drawbacks. Two key aspects are examined for the validation of the proposed approach. First, the effectiveness of the measurement technique in retrieving strain is studied by carrying out four-point bending tests on a full-scale RC beam specimen instrumented with smart cement-based sensors embedded prior to casting. Later, the approach is employed to monitor an RC plate specimen embedding smart cement-based sensors under changing environmental conditions, thus investigating its usefulness for long-term SHM applications.

The paper is organized as follows. Section 2 introduces the proposed distributed biphasic multichannel strain measurement technique also presenting some meaningful preliminary electrical tests. Section 3 describes the concrete structural elements and the type of smart cement-based sensors used in the experiments performed for validating the proposed approach. In the same section, the methodology of each test is also reported in detail. The results obtained in the validation of the measurement technique are illustrated in Section 4, while comments and final remarks are reported in Section 5.

## 2. Multichannel Strain Measurement Technique

**Figure 1:**
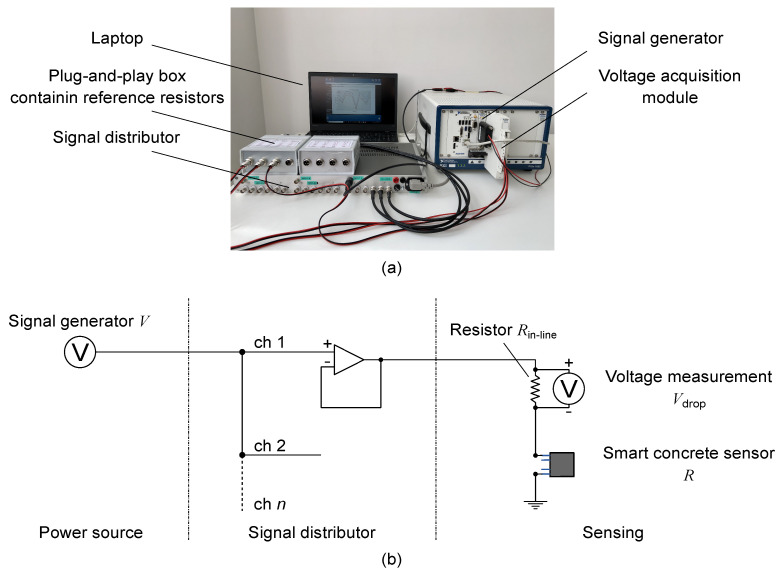
The proposed multichannel strain measurement technique for nanomodified smart cement-based sensors embedded in reinforced concrete structures: (**a**) Example of configuration of the data acquisition and processing system; (**b**) electromechanical diagram exemplifying the proposed approach.

The proposed measurement technique for retrieving strain from nanomodified smart cement-based sensors is designed to ensure the management of multiple independent channels, simultaneous and time-stable inputs and outputs, quick and tidy wiring, and a variety of easy arrangements of data acquisition and processing tasks. An example of setup configuration is shown in Figure 1a, while Figure 1b reports the electromechanical diagram exemplifying the approach. A signal generator is used to power a set of smart cement-based sensors by replicating its voltage input signal on each channel of the sensor array by means of a signal distributor equipped with multiple outputs and designed by the authors at the Laboratory of Structural Dynamics of the University of Perugia (UniPg LabDyn). Each output of the signal distributor, therefore, drives a channel of the sensor array consisting of a smart cement-based sensor placed in series with a reference resistor. Plug-and-play boxes, grouping four channels per box, are used to contain the reference resistors, thus ensuring an orderly wiring. For a single channel, each box provides one input to receive the driving signal from the distributor and two outputs, one to transfer the driving signal to the corresponding smart cement-based sensor and one connected to a multichannel voltmeter that measures the voltage drop at the reference resistor with a specific sample rate. According to this setting, the current, *I*, flowing in the *n*-th channel of the sensor array can be computed as follows:

(1) $$I_{n}=\frac{V_{\mathrm{drop},n}}{R_{\mathrm{in}-\mathrm{line},n}},$$

where $V_{\mathrm{drop},n}$ is the voltage drop measured across the reference resistance, $R_{\mathrm{in}-\mathrm{line},n}$. The electrical resistance of the *n*-th smart cement-based sensor, $R_{n}$, can be determined as follows:

(2) $$R_{n}=\frac{V-V_{\mathrm{drop},n}}{I_{n}},$$

where *V* is the voltage input signal provided by the signal generator. Strain measurements can be retrieved from each smart cement-based sensor included in the network through the processing of its electrical outputs with the following equation:

(3) $$\frac{R-R_{0}}{R_{0}}=\frac{\Delta R}{R_{0}}=\lambda \epsilon ,$$

where *R* and $R_{0}$ are the strained and unstrained electrical resistance of the sensor, respectively, λ is the gauge factor of the sensor, and ϵ is the axial strain (negative in compression).

**Figure 2:**
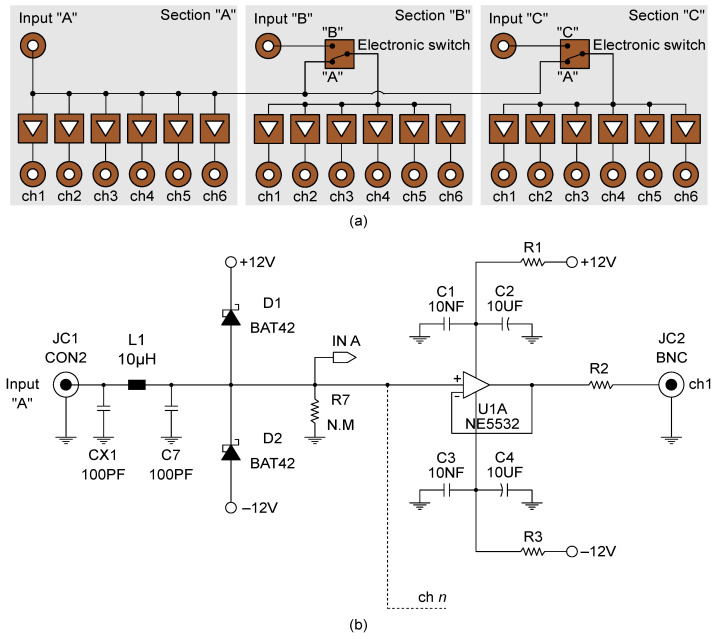
The signal distributor developed at the Laboratory of Structural Dynamics of the University of Perugia (UniPg LabDyn): (**a**) Adopted configuration of the distributor enabling the driving of a maximum of eighteen smart cement-based sensors with the same electrical input; (**b**) example of wiring diagram of a channel of the distributor.

The prototype of the signal distributor used in this work consisted of a compact chassis made up of three independent sections, named “A”, “B”, and “C”, each capable of receiving an input signal from a power source and distributing it on six outputs. In particular, the experiments, hereinafter presented, were performed by setting the signal distributor according to the block diagram shown in Figure 2a, thus allowing the simultaneous driving of a maximum of eighteen outputs in which the unique voltage input signal provided to Section A was replicated. Figure 2b reports the wiring diagram of a channel. As shown, the signal distributor uses Schottky diodes to protect the input against possible voltage spikes and a unity gain buffer, with high input impedance and low output impedance, to ensure the isolation of each channel from the electrical loads in the others. Group delay less than 100 nS, total harmonic distortion less than 0.005%, and signal/noise ratio greater than 96 dB (up to 100 Hz), are other noteworthy features of the adopted signal distributor. A power source module, model NI PXIe-4138, and a multichannel analog input module, model NI PXIe-4302, both mounted on a chassis, model NI PXIe-1092, were used to provide a voltage square wave input of 20 V peak-to-peak, duty cycle of 50%, and frequency of 1 Hz, and to perform accurate voltage measurements at the reference resistors with a sample rate of 10 Hz, respectively. It should be noted that any other signal generator and voltmeter with similar characteristics to those mentioned can be implemented in the approach. Likewise, the typology of the voltage input signal can be modified (e.g., employing AC or DC voltage input). Here, the adopted biphasic DC voltage input signal cyclically charges and discharges the sensors eliminating any undesired time drift caused by the polarization effect (as subsequently demonstrated). Accordingly, the electrical resistance of the *n*-th smart cement-based sensor, $R_{n}$, was computed by considering the positive plateau of the voltage input signal, *V*, equal to 10 V and by sampling discrete voltage measurements in correspondence of the 80% of the cyclic positive constant part of the voltage intensity output acquired at the corresponding reference resistor, thus obtaining values of the electrical resistance with the same sample rate of the voltage input signal, i.e., 1 Hz [26]. Data acquisition and processing were automated by using a dedicated software written in Python.

### 2.1. Input–Output Simultaneity Assessment

The simultaneous management of multiple independent channels represents the strength of the proposed strain measurement technique. In light of that, electrical tests were carried out to prove the input–output simultaneity of the adopted signal distributor and the analog input module. Being focused on testing the instrumentation, experiments were performed by considering a setup involving seven channels not connected to smart cement-based sensors. A first test was conducted by powering the channels with a sinusoidal waveform input of 20 V peak-to-peak, duty cycle of 50%, and frequency of 1 Hz, measuring the voltage drops at the reference resistors for one second with a constant sample rate set at 5000 Hz (the maximum value allowed by the adopted multichannel analog input module). Then, a second test was performed by increasing the frequency of the voltage input signal to 10 Hz.

**Figure 3:**
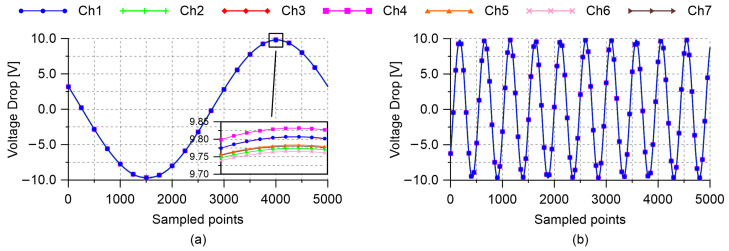
Voltage measurements recorded from multiple channels by carrying out preliminary electrical tests on the instrumentation. Acquisitions were performed for one second with a sample rate of 5000 Hz: (**a**) Sinusoidal waveform input of 20 V peak-to-peak, duty cycle of 50%, and frequency of 1 Hz; (**b**) sinusoidal waveform input of 20 V peak-to-peak, duty cycle of 50%, and frequency of 10 Hz.

The voltage measurements recorded by carrying out the first and the second test are reported in Figure 3a,b, respectively. Both the pictures show synchronized signals without relevant shape alterations, noise nor delays, thus confirming that both the signal distributor and the multichannel analog input module are capable of operating on multiple independent channels simultaneously.

### 2.2. Stability of the Electrical Measurements

**Figure 4:**
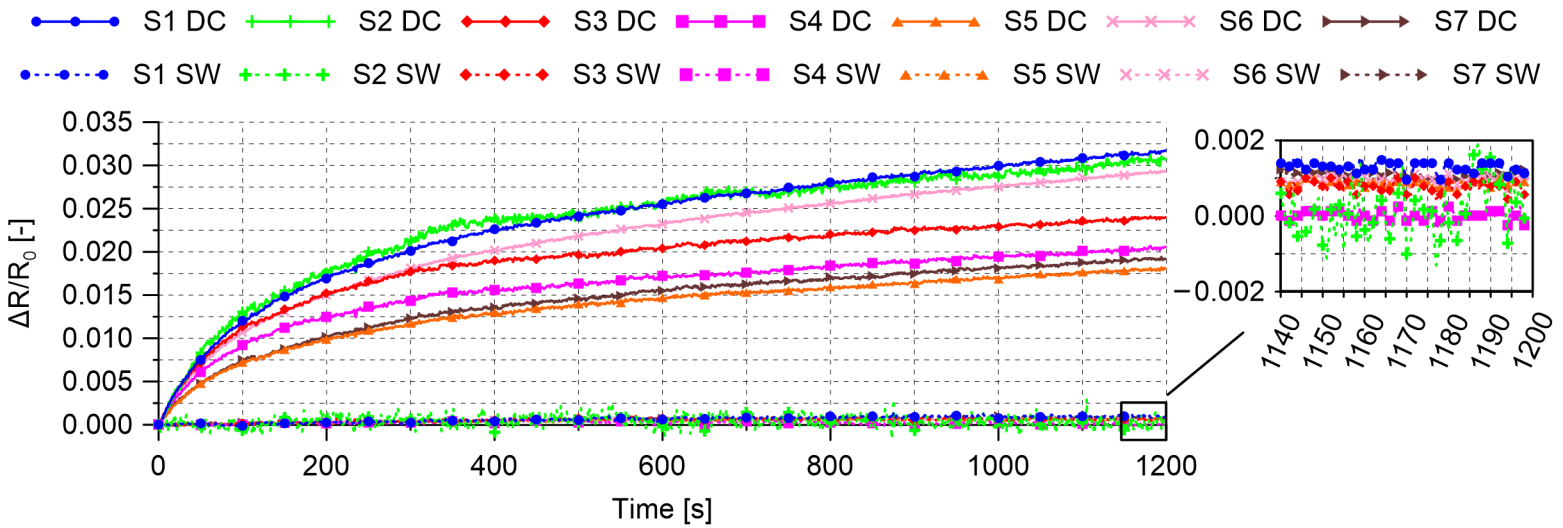
Trends of the relative change in electrical resistance of the smart cement-based sensors obtained by using a DC voltage (DC) input and a voltage square wave (SW) input to perform electrical measurements in unloaded conditions with the proposed new measurement technique.

Electrical tests were carried out also to evaluate the stability of the electrical measurements performed with the proposed measurement technique in unloaded conditions by using different typologies of voltage input signals. In particular, a first test was carried out by employing a DC voltage input of 10 V to perform electrical measurements on a set of seven smart cement-based sensors for 20 min. Then, the experiment was repeated, on the same samples for the same time, by using a voltage square wave input of 20 V peak-to-peak, duty cycle of 50%, and frequency of 1 Hz. In both cases, the voltage drops at the reference resistors were acquired with a constant sample rate of 10 Hz, then processed through Equation (2) to compute the electrical resistance of the adopted sensors. The results obtained in these tests are shown in Figure 4, which clearly points out the optimal performance of the distributed voltage square wave input, the use of which allowed to remove any polarization effect and to eliminate any time drift in the trends of the relative change in electrical resistance of the smart cement-based sensors. The figure also demonstrates that all sensors were characterized by a similar electrical behavior and noise level, thus confirming the feasibility of the measurement procedure.

## 3. Methodology for Validation

This section outlines the validation of the proposed multichannel strain measurements technique. Details concerning the tested full-scale structural elements and the setup of the sensor networks, including calibration sensors, are first provided. Then, the methodologies adopted for conducting the experiments are illustrated.

### 3.1. Tested Specimens

**Figure 5:**
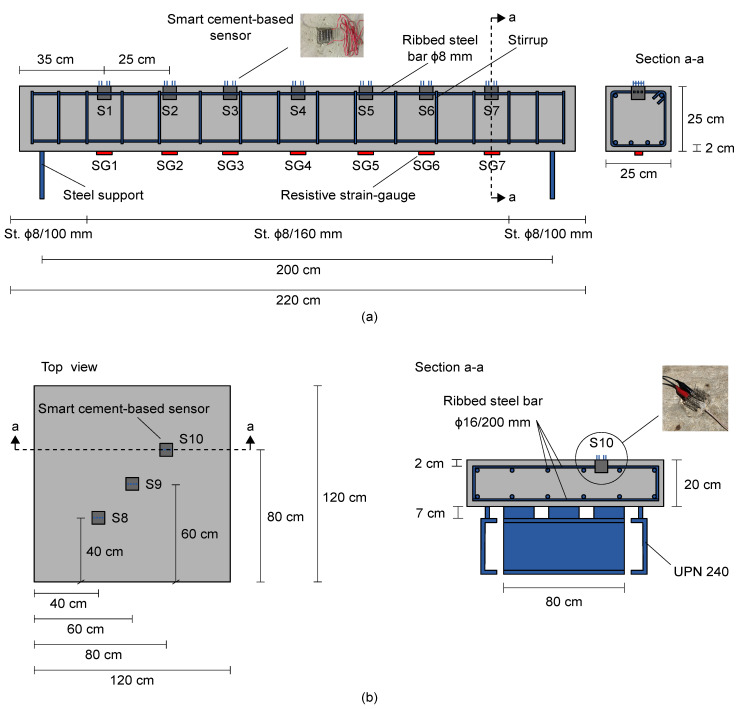
Graphical representations of the reinforced concrete structural elements tested for validating the proposed multichannel strain measurement technique: (**a**) Beam specimen subjected to four-point bending tests with annotated the main construction details and the deployment of smart cement-based sensors and resistive strain-gauges; (**b**) plate specimen tested under changing environmental effects with annotated the main construction details and the deployment of smart cement-based sensors.

The main construction details of the simply supported RC beam specimen subjected to four-point bending tests are shown in Figure 5a, together with the deployment of the smart cement-based sensors and resistive strain-gauges used to measure compressive and tensile strain states, respectively, in the structural element. The smart cement-based sensors were cube samples of 5 cm side equipped with four stainless steel mesh electrodes to perform electrical measurements. Manufactured by doping a cementitious matrix of cement paste with the 1% of MWCNTs with respect to the weight of cement, such sensors were embedded within the beam specimen in September 2016, while 60 mm long strain-gauges, model Kyowa KC-60-120-A1-11L1M2R, were externally attached to the bottom surface of the structural element before testing. As better detailed in the following developments of the paper, strain gauges were used for a post-embedding calibration of the smart cement-based sensors. It should be noted that in the case of the tested beam specimen, the number and locations of the two types of sensors did not necessarily need to be the same for performing such a calibration procedure; therefore, two different positions for smart sensors and strain gauges (extrados and intrados, respectively) were chosen for illustrative purposes. Yet, a similar approach requires a reliable model relating strain values at the locations of the cement-based sensors to those at the locations of the strain-gauges. When such a reliable model is available, a similar approach is also applicable in the field. Otherwise, the initial calibration of the smart sensors may require the application of strain-gauges in their proximity to effectively measure their actual compressive strain state.

Figure 5b shows a conceptual illustration of the RC plate specimen tested under changing environmental conditions. It consisted of a thick concrete plate of square shape supported by a steel support system and reinforced with twenty-four ribbed steel bars with a diameter of 16 mm, deployed along with the two main directions by forming a grid. The structural element was instrumented with three smart cement-based sensors belonging to the same set as those embedded within the beam specimen. Similar to the latter, the plate specimen was also built in September 2016 by using a commercial premixed concrete type “Concrete Rck30” by Colacem (Italy), with a maximum diameter of the siliceous aggregates of 15 mm and a water/cement ratio of 0.55. It is worth pointing out that nanomodified cement paste sensors were adopted to instrument the described RC specimens since the absence of aggregates within their cementitious matrices contributes to reducing the noise level in their electrical outputs and results in optimal strain sensing properties [18]. Furthermore, their cubic shape was chosen to facilitate sensors fabrication using standard molds. Although both the use of nanomodified cement paste and the choice of a cubic shape are relatively common in the literature [29,30], it should be mentioned that the mechanical compatibility between nanomodified cement paste and structural concrete, as well as its effects over measured strain, would deserve further studies and that the shape of the strain sensors may be optimized to reduce the risk of concrete cracking due to presence of notches associated to the cubic shape of the sensors. These aspects are however out of the scope of the present study. Further details about the smart sensors, including information on their production process, can be found in D’Alessandro et al. [20].

### 3.2. Four-Point Bending Tests on an RC Beam Specimen

**Figure 6:**
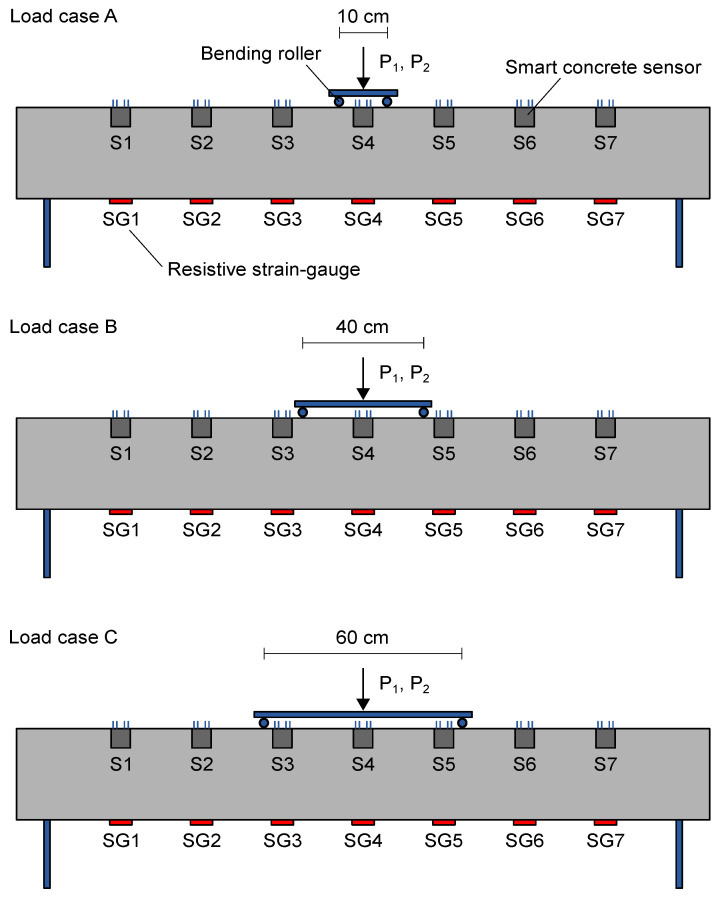
Load cases considered for carrying out four-point bending tests on the reinforced concrete beam specimen (P${}_{1}$ and P${}_{2}$ indicate two consecutive loads of different value applied on the structural element).

The effectiveness of the proposed multichannel approach in retrieving strain measurements from nanomodified smart cement-based sensors was investigated by carrying out four-point bending tests on the RC beam specimen, then by using the output obtained from each embedded sensor to reconstruct the strain distribution within the structural element under different load cases. In particular, tests were conducted by considering three different settings, named A, B, and C, in which the position of the bending rollers of the loading structure varied as reported in Figure 6. Each load case was performed first by applying a load of magnitude of 2 kN, hereinafter referred to as P${}_{1}$, then a load equal to 3 kN, named P${}_{2}$. The entity of the applied loads was kept low to maintain unaltered the structural integrity of the beam specimen and to test the measurement technique in challenging conditions associated with small changes in strain. Electrical measurements were carried out on the embedded sensors after each application of the load. Unstrained electrical signals were also recorded from the sensors in unloading conditions of the beam specimen. Strain measurements were therefore retrieved by processing these electrical outputs according to Equation (3), set by determining the gauge factors of the sensors integrated within the beam specimen through a post-embedding calibration algorithm described in the following section.

#### Post-Embedding Calibration Algorithm for Smart Cement-Based Sensors Integrated in RC Beams

**Figure 7:**
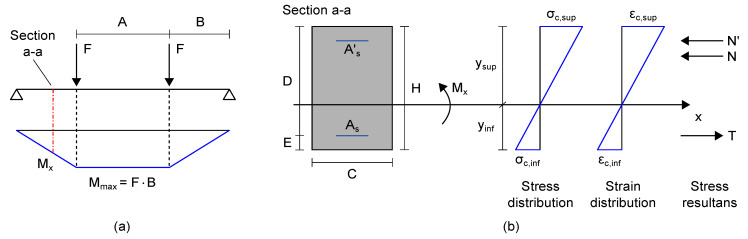
Analytical models considered for defining the post-embedding calibration algorithm for smart cement-based sensors embedded in reinforced concrete (RC) beams: (**a**) Bending moment diagram for a simply supported beam subjected to two concentrated loads, F (where M${}_{max}$ is the maximum value of the bending moment and M${}_{x}$ is the bending moment acting on a section of the beam specimen instrumented with a smart cement-based sensor); (**b**) stress and strain diagrams for the uncracked concrete stage theory in a generic RC section (x is the neutral axis, H is the height of the section, E indicates the concrete cover, D is defined as the difference between H and E).

Strain measurements can be retrieved through the processing of the electrical outputs from nanomodified smart cement-based sensors by carrying out their initial calibration after their embedding in an RC structure to take into account the actual boundary conditions of each sensor within the load-bearing structure. In light of that, a post-embedding calibration algorithm was defined to retrieve the gauge factor of each sensor embedded within the RC beam specimen by taking into account the load case A and the application of the load P${}_{1}$ as reference conditions. Measurements from the strain-gauges glued to the structural element, acquired through a data acquisition system, model IMC Cronos-PL 16, set with a sampling frequency of 1 Hz and a quarter-bridge configuration with a nominal gauge resistance of 120 Ω, were also considered in the algorithm. First of all, the bending moment, M${}_{x}$, acting on each section of the beam specimen instrumented with a smart cement-based sensor was determined according to the static scheme of simply supported beam depicted in Figure 7a. Then, the equivalent average Young Modulus of the concrete composing the structural element, E${}_{c}$, was iteratively estimated by considering the uncracked concrete stage theory (Figure 7b) in which the analytical tensile strain, $\epsilon_{c,\inf}$, at the mid-span of the beam specimen was imposed to be equal to the strain measured by the strain-gauge named SG4. The material properties and mechanical parameters of the structural element considered in this computation are collected in Table 1. Here, the equivalent Young Modulus of concrete, E${}_{c}$, retrieved in the final iteration is reported. Its relatively small value may be attributed to epistemic uncertainties, given the very simple assumed linear uncracked beam model, condition determined by the low loads applied during the tests. Exploiting this parameter and once again the uncracked concrete stage theory, both the analytical tensile and compressive strains were determined at each section of the beam specimen instrumented with a smart cement-based sensor. Accordingly, the gauge factor of a smart cement-based sensor embedded in the structural element was retrieved from Equation (3) by taking into account the relative change in electrical resistance, $\Delta R/R_{0}$, from the sensor, and the corresponding compressive strain, $\epsilon_{c,\sup}$, from the analytical model. It is worth noting that the use of the experimental value of the Young Modulus of on-site cured concrete would simplify the proposed post-embedding calibration procedure and, more importantly, would reduce the epistemic uncertainties otherwise introduced in the estimation of the gauge factors of the smart cement-based sensors. In the absence of such an experimental value, the equivalent average Young Modulus of the concrete iteratively estimated through the proposed procedure can be considered representative of the actual behavior of the structural element and thus adequate for estimating the gauge factors of the sensors, albeit with some approximations.

### 3.3. RC Plate Specimen Subjected to Changing Environmental Conditions

**Figure 8:**
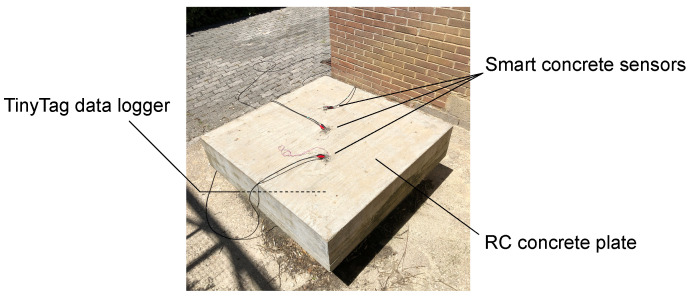
Field application of the multichannel strain measurement technique for monitoring a reinforced concrete plate specimen embedding smart cement-based sensors under environmental effects (variations in temperature and humidity).

The multichannel strain measurement technique was field-tested by considering its application for carrying out electrical measurements on the smart cement-based sensors monitoring the RC plate specimen under environmental effects (Figure 8). This structural setting would exemplify the long-term SHM of an RC structure where changes in the electrical outputs of the sensors due to the variability of temperature and humidity over time need to be properly characterized for compensation purposes. This aspect is crucial for the retrievement of unbiased strain measurements representative only of the changes in the structural setting (e.g., application of external loads), which often result in variations in the electrical outputs of the smart cement-based sensors of smaller entity than those due to the changes in the environmental conditions. In light of that, this field application is focused on demonstrating the effectiveness of the proposed technique in simultaneously measuring the time-based change induced in the electrical output of each sensor by the environmental effects for its subsequent removal. The experiment was conducted by performing continuous electrical measurements on the sensors embedded in the plate specimen for three days, by carrying out six measurements per hour. The hourly trend of the electrical resistance of each sensor was reconstructed by computing the average of the records within each hour. With a similar methodology, changes in the air temperature and relative humidity were recorded by means of a TinyTag sensor, model TGP-4500, from Gemini Data Loggers, positioned under the plate specimen to avoid its direct exposure to the sun.

## 4. Results

This section collects the results achieved in the experiments carried out for the validation of the proposed multichannel strain measurement technique.

### 4.1. Four-Point Bending Tests on an RC Beam Specimen

**Figure 9:**
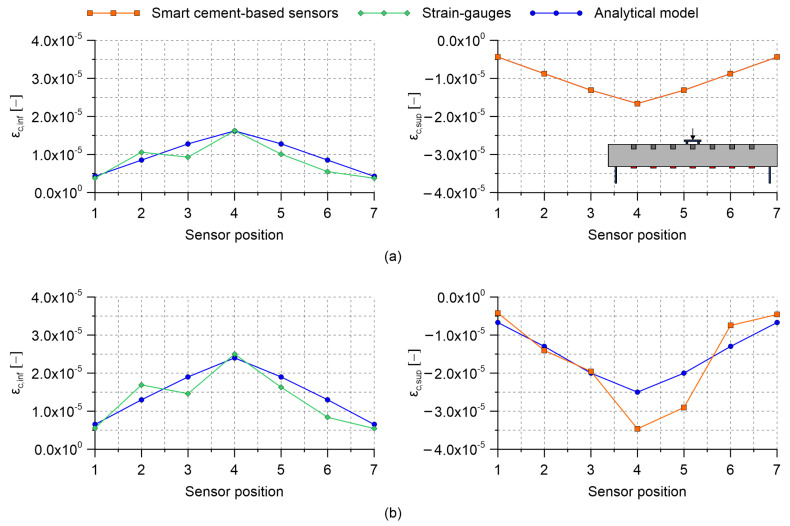
Distribution of the tensile and compressive strain obtained by carrying out four-point bending tests on the reinforced concrete beam specimen embedding smart cement-based sensors (where $\epsilon_{c,\inf}$ and $\epsilon_{c,\sup}$ are the tensile and compressive strain within a considered section of the beam, respectively): (**a**) Load case A, applied load P${}_{1}$ = 2 kN; (**b**) load case A, applied load P${}_{2}$ = 3 kN.

**Figure 10:**
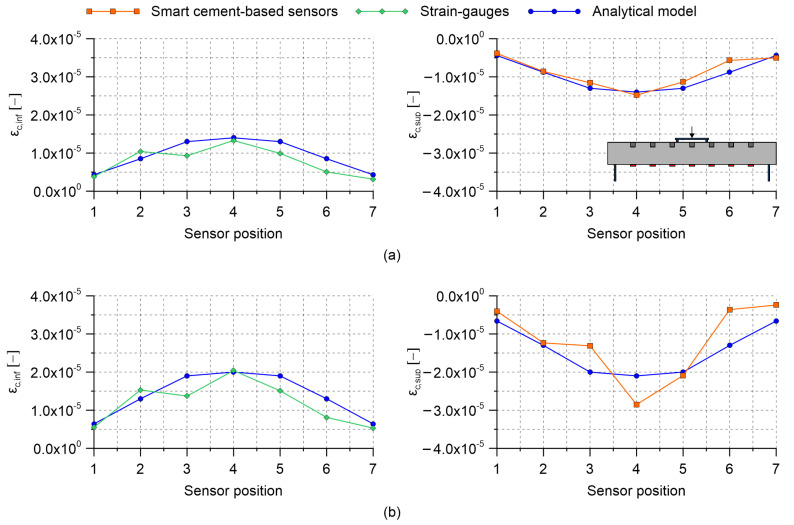
Distribution of the tensile and compressive strain obtained by carrying out four-point bending tests on the reinforced concrete beam specimen embedding smart cement-based sensors (where $\epsilon_{c,\inf}$ and $\epsilon_{c,\sup}$ are the tensile and compressive strain within a considered section of the beam, respectively): (**a**) Load case B, applied load P${}_{1}$ = 2 kN; (**b**) load case B, applied load P${}_{2}$ = 3 kN.

**Figure 11:**
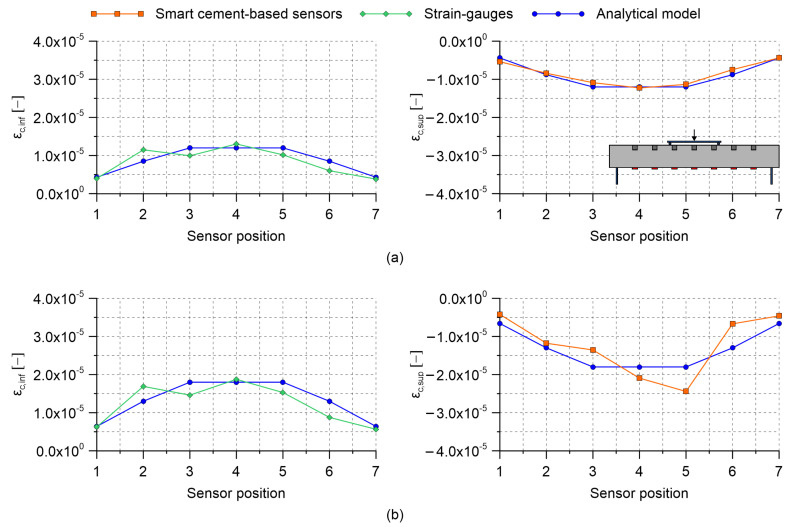
Distribution of the tensile and compressive strain obtained by carrying out four-point bending tests on the reinforced concrete beam specimen embedding smart cement-based sensors (where $\epsilon_{c,\inf}$ and $\epsilon_{c,\sup}$ are the tensile and compressive strain within a considered section of the beam, respectively): (**a**) Load case C, applied load P${}_{1}$ = 2 kN; (**b**) load case C, applied load P${}_{2}$ = 3 kN.

**Figure 12:**
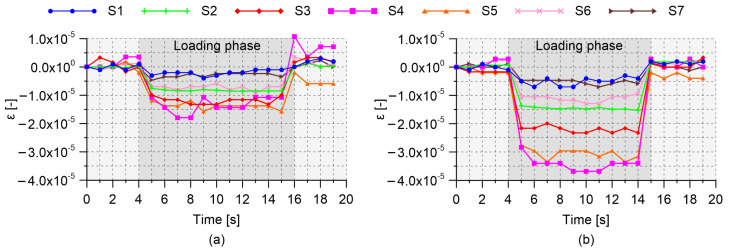
Trends of the strain retrieved from the smart cement-based sensors embedded in the reinforced concrete beam specimen by carrying out four-point bending tests: (**a**) Load case A, applied load P${}_{1}$ = 2 kN; (**b**) load case A, applied load P${}_{2}$ = 3 kN.

Results obtained by carrying out four-point bending tests on the RC beam specimen with embedded nanomodified smart cement-based sensors are herein presented. Table 2 collects the gauge factors estimated through the post-embedding calibration algorithm. Almost all the obtained values are within the same range of magnitude indicating that the embedded sensors possessed similar strain-sensing capabilities, except for the one named S2, which was characterized by a gauge factor significantly higher than the others. Examining the origin of such a different gauge factor goes beyond the purposes of the present work; however, the superior sensitivity to the applied strain shown by sensor S2 can be conceivably attributed to a better material continuity with the beam specimen, which therefore resulted in a more effective exchange of stresses compared to what happened between the structural element and the other embedded sensors. This aspect remarks the importance of calibrating the smart sensors after their embedding within a structural element to take into account their actual boundary conditions. Figure 9, Figure 10 and Figure 11 report the distributions of the tensile and compressive strain within the beam specimen obtained from strain-gauges and smart cement-based sensors, respectively, together with the strains computed through the uncracked concrete stage theory. These plots point out a satisfactory agreement between the measurements from strain-gauges and those from the analytical model, although with some conceivable approximations, thus confirming the effectiveness of the latter in describing the strain state of the beam specimen under the performed load cases. Accordingly, compressive strains obtained through the uncracked concrete stage theory can be reasonably employed as a benchmark for the validation of those retrieved from the smart cement-based sensors. In this regard, it is worth noting that the distributions of the compressive strain within the beam specimen obtained through the measurements from the embedded sensors are in general consistent with those obtained from the analytical model. Figure 9a shows values of the tensile and compressive strain obtained by applying the load P${}_{1}$ on the beam specimen set according to the load case A. Since this setting was considered for the post-embedding calibration of the smart cement-based sensors, their compressive strains perfectly match the analytical ones computed with the uncracked concrete stage theory. For the same reason, the analytical tensile strain calculated at the midspan of the beam specimen matches the output of the strain-gauge named SG4. Figure 9b shows the distribution of the strain within the beam specimen after the application of the load P${}_{2}$. Consistently, an increment in the magnitude of the strains can be noted both in tension and in compression with respect to the previous application of the load P${}_{1}$. Moreover, the plot highlights that the distribution of the compressive strain provided by the smart cement-based sensors differed slightly from that obtained from the uncracked concrete stage theory, indicating that greater strains occurred in correspondence of the embedded sensors named S4 and S5. Of particular interest is the comparison between the plots relating to the load case A and those shown in Figure 10 for the load case B. In both the settings the maximum value of the strain was expected in correspondence of the mid-span of the beam specimen, with strains of smaller entity measured in the load case B than in A due to the employment of more spaced bending rollers. Coherently to this, in load case B the embedded sensors outputted strain values lower than those measured in load case A, pointing out that the mid-span section of the beam specimen was the most compressed in both the settings. Similarly to load case A, Figure 10a,b show that the application of the load P${}_{2}$ on the beam specimen, set according to the load case B, produced an increase in the compressive strain measured with the smart cement-based sensors with respect to the application of the load P${}_{1}$, by inducing a strain concentration in the proximity of sensors labeled S4 and S5. Results obtained by testing the beam specimen according to the load case C are reported in Figure 11. Here, the portion of the structural element subjected to the maximum value of the bending moment was further extended compared to the load cases A and B. Consequently, the smart cement-based sensors deployed in that area, i.e., S3, S4, and S5, coherently provided strains of similar value during the application of the load P${}_{1}$, while the application of the load P${}_{2}$ induced a general increment in strain values together with a slight strain concentration within the embedded sensors named S4 and S5. As expected, compressive strains retrieved from the smart cement-based sensors were smaller than those obtained for load cases A and B due to the smaller bending moment. Figure 12a,b report the trends of the strain provided by the smart cement-based sensors by applying the loads P${}_{1}$ and P${}_{2}$ on the beam specimen, respectively, for the load case A. These graphs clearly point out that the acquisition and processing of the electrical outputs of the smart cement-based sensors through the proposed approach allowed retrieving time stable strain measurements under the application of the considered static loads. The graphs also show that S2 sensor provided the most stable strain measurement among the whole sensor array, thus further supporting the hypothesis that this smart sensor was subject to a greater initial compression compared to the others. Similar results were found also for the other load cases, however, their discussion is omitted herein for the sake of brevity.

### 4.2. RC Plate Specimen Subjected to Changing Environmental Conditions

**Figure 13:**
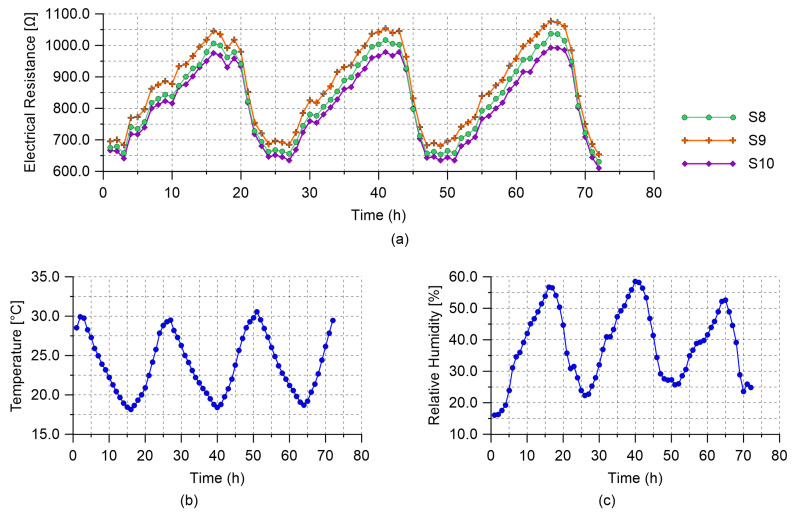
Results from the monitoring of the reinforced concrete plate specimen subjected to environmental effects: (**a**) Hourly trends of the electrical resistance measured from the smart cement-based sensors embedded within the plate specimen; (**b**) hourly trend of the air temperature; (**c**) hourly trend of the relative humidity.

**Figure 14:**
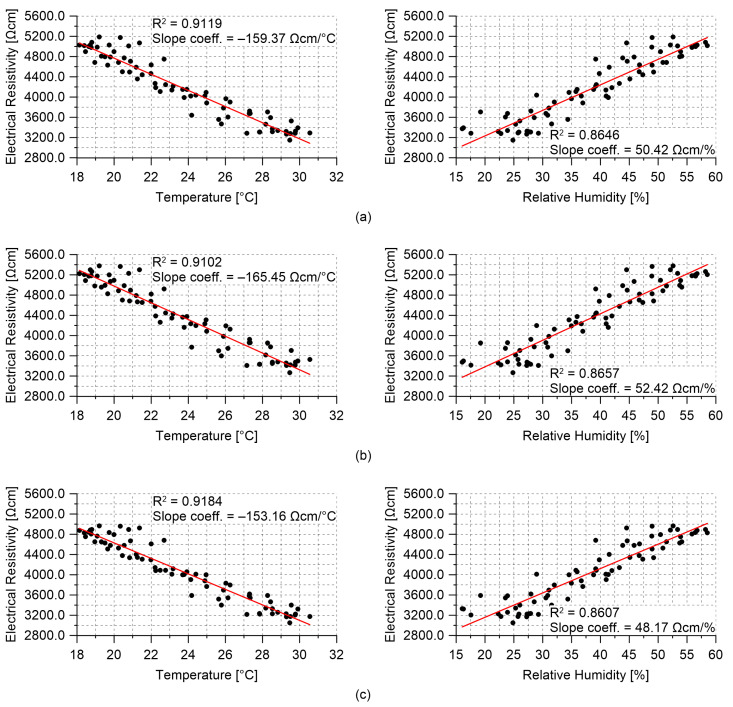
Electrical resistivity computed from the outputs of the smart cement-based sensors embedded within the reinforced concrete plate specimen versus air temperature and relative humidity data: (**a**) S8 sensor; (**b**) S9 sensor; (**c**) S10 sensor.

Results obtained from the field application of the multichannel strain measurement technique are presented in this section. Figure 13 reports the hourly trends of the electrical resistance measured from the nanomodified smart cement-based sensors embedded within the RC plate specimen, together with the trends of the air temperature and relative humidity recorded by the TinyTag data logger. The electrical outputs in Figure 13a appear synchronous with each other and coherent with the temperature measurements plotted in Figure 13b. In particular, the plots show that a decrease in temperature resulted in an increase in the value of the electrical resistance of all the embedded sensors. This phenomenon demonstrates that the smart cement-based sensors electrically behave as semi-conductive materials when cooled or heated although only a tailored investigation could characterize in detail the physical properties of these types of novel materials. In this regard, the assessment of the phase shift between the outputs from the smart sensors and the measurements of the air temperature due to concrete thermal storage capacity deserves special attention and will be the subject of future studies. On the other hand, variations in humidity seem to represent a secondary effect with respect to the influence of the temperature on the electrical outputs. The effects of the changes in the environmental conditions can be compensated from the electrical outputs of the sensors by leveraging on the linear correlation, shown in Figure 14, which characterizes the data. This last graph also points out that the employed sensors were characterized by a similar sensitivity to the changes in temperature since slope coefficients of similar values were determined from the linear regression analysis carried out on data of the electrical resistivity computed for each sensor and of the temperature. Likewise, it can be noted that sensors possessed comparable sensitivities also to the changes in humidity, although the temperature–humidity correlation is conceivably the driver of such resistance–humidity correlation. The consistency between the outputs of the different sensors under changing environmental conditions suggests that a similar compensation strategy can be used for all of them, which is crucial for SHM applications.

## 5. Conclusions

The paper proposed a multichannel strain measurement technique for nanomodified smart cement-based sensors embedded in RC structures especially conceived to allow simultaneous and time-stable strain measurements from an arbitrary number of independent sensors. The methodology and the results obtained in the experimental campaign carried out to test its effectiveness were also presented.

An RC beam specimen instrumented with a fully integrated array of smart cement-based sensors was subjected to four-point bending tests to study the effectiveness of the proposed measurement technique in retrieving strains through the processing of electrical outputs acquired from smart cement-based sensors embedded in an RC structure. Tests were conducted by considering different load cases with the aim of varying the entity of bending moment acting on the beam specimen, using the embedded sensors to reconstruct the distribution of the compressive strain within the structural element. A simple analytical model, based on the uncracked concrete stage theory, was adopted to validate the strains retrieved through the proposed approach. In this experiment, a post-embedding calibration procedure for embedded smart cement-based sensors was also defined and adopted to determine the gauge factor of the sensors integrated within the beam specimen. Moreover, a RC plate specimen with a set of embedded smart cement-based sensors was monitored under changing environmental conditions simulating a field application of the multichannel strain measurement technique for long-term SHM. This experiment was aimed at demonstrating the advantages of performing simultaneous electrical measurements on multiple embedded sensors for the assessment of the influence of changes in temperature and humidity on their electrical outputs.

The results obtained by testing the beam specimen under bending loads showed that the strain measurements retrieved with the multichannel strain measurement technique were consistent with all the investigated load cases and with the strains computed by means of the analytical model. Accordingly, this experimentation successfully demonstrated the effectiveness of the proposed measurement technique in retrieving time-stable strain from smart cement-based sensors embedded in an RC structure. Not least, the bending tests also pointed out the usefulness of the post-embedding calibration procedure proposed for the determination of the gauge factor of smart cement-based sensors a long time after their embedding within RC beams. It is worth highlighting that being in principle adaptable to concrete elements characterized by different static schemes, the investigated calibration approach could be also effective in practical SHM applications; however, further studies are needed to fully prove its effectiveness. The results obtained in the field application pointed out that that the employment of multichannel strain measurement technique for long-term SHM of RC structures can be particularly promising. In particular, the experiment highlighted the capability of the measurement technique in carrying out simultaneous readings from multiple smart cement-based sensors under varying environmental conditions. As temperature and humidity are variable over time, the proposed sensing system allowed the contemporary evaluation of the time-based changes induced by such environmental effects in the electrical outputs acquired from the sensors embedded in the plate specimen. In this context, the test also highlighted that the electrical resistance of the smart cement-based sensors is mostly affected by the hourly variations in temperature and that all the sensors exhibit consistent values of temperature sensitivity.

Overall, it is concluded that the multichannel strain measurement technique proposed and validated in this work represents a promising approach for performing feasible field implementations of smart cement-based sensors to RC structures. In this regard, further studies considering field applications to full-scale concrete structures are needed first to conduct a significant error analysis of the proposed measurement technique, then to demonstrate its effectiveness for the long-term monitoring of the static response of RC structures during their service life, including a deep understanding and modeling of the effects of changing environmental conditions over the electrical response of the smart sensors, as well as an investigation on their use at relatively high compression states.

## Figures and Tables

**Table 1:** Material properties and mechanical parameters of the reinforced concrete beam specimen: Area of the reinforcing steel bar under tension, A${}_{s}$, area of the reinforcing steel bar under compression, ${A^{\prime }}_{s}$, Young Modulus of reinforcing steel bar, E${}_{s}$, equivalent Young Modulus of concrete, E${}_{c}$, homogenization coefficient, n.

Reinforcing Steel Bar B450C		Concrete Rck30	
A${}_{s}$	200 mm${}^{2}$	E${}_{c}$	20,750 MPa
A${}^{{}^{\prime }}$${}_{s}$	100 mm${}^{2}$	n = E${}_{s}$/E${}_{c}$	10.12
E${}_{s}$	210,000 MPa		

**Table 2:** Gauge factor, λ, estimated for each smart cement-based sensor embedded within the reinforced concrete beam specimen through the proposed post-embedding calibration algorithm.

Sensor	λ	Sensor	λ
S1	59	S5	28
S2	594	S6	39
S3	45	S7	46
S4	28		

## Data Availability

The data that support the findings of this study are available from the corresponding author, A.D., upon reasonable request.

## References

[1] Han B., Yu X., Ou J. (2014). Self-Sensing Concrete in Smart Structures.

[2] D’Alessandro A., Materazzi A.L., Ubertini F. (2020). Nanotechnology in Cement-Based Construction.

[3] American Concrete Institute C. (2021). Structural Health Monitoring Technologies for Concrete Structures—Report.

[4] Konsta-Gdoutos M.S., Aza C.A. (2014). Self sensing carbon nanotube (CNT) and nanofiber (CNF) cementitious composites for real time damage assessment in smart structures. Cem. Concr. Compos..

[5] Han B., Wang Y., Dong S., Zhang L., Ding S., Yu X., Ou J. (2015). Smart concretes and structures: A review. J. Intell. Mater. Syst. Struct..

[6] Camacho-Ballesta C., Zornoza E., Garcés P. (2016). Performance of cement-based sensors with CNT for strain sensing. Adv. Cem. Res..

[7] Hou T.C., Lynch J.P. (2009). Electrical impedance tomographic methods for sensing strain fields and crack damage in cementitious structures. J. Intell. Mater. Syst. Struct..

[8] Han B., Yu X., Ou J. (2011). Multifunctional and smart carbon nanotube reinforced cement-based materials. Nanotechnology in Civil Infrastructure.

[9] Rana S., Subramani P., Fangueiro R., Correia A.G. (2016). A review on smart self-sensing composite materials for civil engineering applications. AIMS Mater. Sci..

[10] Birgin H., D’Alessandro A., Ubertini F. (2020). Smart Graphite–Cement Composite for Roadway-Integrated Weigh-In-Motion Sensing. Sensors.

[11] Shah S.P., Konsta-Gdoutos M., Metaxa Z., Mondal P. (2009). Nanoscale modification of cementitious materials. Nanotechnology in Construction 3.

[12] Azhari F., Banthia N. (2012). Cement-based sensors with carbon fibers and carbon nanotubes for piezoresistive sensing. Cem. Concr. Compos..

[13] Galao O., Baeza F.J., Zornoza E., Garcés P. (2014). Strain and damage sensing properties on multifunctional cement composites with CNF admixture. Cem. Concr. Compos..

[14] Yang Q., Liu P., Ge Z., Wang D. (2020). Self-Sensing Carbon Nanotube-Cement Composite Material for Structural Health Monitoring of Pavements. J. Test. Eval..

[15] Baeza F., Zornoza E., Andión L.G., Ivorra S., Garcés P. (2011). Variables affecting strain sensing function in cementitious composites with carbon fibers. Comput. Concr..

[16] Coppola L., Buoso A., Corazza F. (2011). Electrical properties of carbon nanotubes cement composites for monitoring stress conditions in concrete structures. Appl. Mech. Mater..

[17] D’Alessandro A., Rallini M., Ubertini F., Materazzi A.L., Kenny J.M. (2016). Investigations on scalable fabrication procedures for self-sensing carbon nanotube cement-matrix composites for SHM applications. Cem. Concr. Compos..

[18] Meoni A., D’Alessandro A., Downey A., García-Macías E., Rallini M., Materazzi A., Torre L., Laflamme S., Castro-Triguero R., Ubertini F. (2018). An experimental study on static and dynamic strain sensitivity of embeddable smart concrete sensors doped with carbon nanotubes for SHM of large structures. Sensors.

[19] Saafi M. (2009). Wireless and embedded carbon nanotube networks for damage detection in concrete structures. Nanotechnology.

[20] D’Alessandro A., Ubertini F., García-Macías E., Castro-Triguero R., Downey A., Laflamme S., Meoni A., Materazzi A.L. (2017). Static and dynamic strain monitoring of reinforced concrete components through embedded carbon nanotube cement-based sensors. Shock Vib..

[21] Ding S., Ruan Y., Yu X., Han B., Ni Y.Q. (2019). Self-monitoring of smart concrete column incorporating CNT/NCB composite fillers modified cementitious sensors. Constr. Build. Mater..

[22] Dong W., Li W., Luo Z., Long G., Vessalas K., Sheng D. (2020). Structural response monitoring of concrete beam under flexural loading using smart carbon black/cement-based sensors. Smart Mater. Struct..

[23] Castañeda-Saldarriaga D.L., Alvarez-Montoya J., Martínez-Tejada V., Sierra-Pérez J. (2021). Toward Structural Health Monitoring of Civil Structures Based on Self-Sensing Concrete Nanocomposites: A Validation in a Reinforced-Concrete Beam. Int. J. Concr. Struct. Mater..

[24] Han B., Guan X., Ou J. (2007). Electrode design, measuring method and data acquisition system of carbon fiber cement paste piezoresistive sensors. Sens. Actuators A Phys..

[25] Machan L., Steffan P. (2015). Multichannel Laboratory Device for Measurement of Smart Concrete Material Properties. Advanced Materials Research.

[26] Downey A., D’Alessandro A., Ubertini F., Laflamme S., Geiger R. (2017). Biphasic DC measurement approach for enhanced measurement stability and multi-channel sampling of self-sensing multi-functional structural materials doped with carbon-based additives. Smart Mater. Struct..

[27] Downey A., D’Alessandro A., Baquera M., García-Macías E., Rolfes D., Ubertini F., Laflamme S., Castro-Triguero R. (2017). Damage detection, localization and quantification in conductive smart concrete structures using a resistor mesh model. Eng. Struct..

[28] Kang M.S., Lee H., Yim H.J., An Y.K., Kim D.J. (2018). Multi-channel electrical impedance-based crack localization of fiber-reinforced cementitious composites under bending conditions. Appl. Sci..

[29] Yu X., Kwon E. (2009). A carbon nanotube/cement composite with piezoresistive properties. Smart Mater. Struct..

[30] Yoo D.Y., You I., Zi G., Lee S.J. (2019). Effects of carbon nanomaterial type and amount on self-sensing capacity of cement paste. Measurement.

